# Recurrent poliserositis succesfully treated with IL-1 receptor antagonist Anakinra

**DOI:** 10.1186/1546-0096-9-S1-P4

**Published:** 2011-09-14

**Authors:** MS Camacho Lovillo, E Iglesias, O Neth, V Santa-María, JL Gavilán

**Affiliations:** 1 Department of Pediatric Infectious Diseases and Immunodeficiencies, Hospital Infantil Virgen del Rocío, Seville, Spain; 2 Department of Pediatric Cardiology, Hospital Infantil Virgen del Rocío, Seville, Spain

## Background

Recurrent pericarditis is known to be a clinical feature in numerous inflammatory and infectious diseases. However, in the majority of cases, recurrent pericarditis appears to be idiopathic. The optimal regimen for preventing recurrence is not established; treatment modalities include non-steroidal anti-inflammatory drugs (NSAIDs), corticosteroids, colchicine and other immunomodulatory agents as well as pericardiectomy. Long-term prognosis of recurrent pericarditis is generally good.

## Case report

A previously healthy twelve years old boy was admitted in March 2008 to our hospital with fever and thoracic pain starting 3 days after a minor road traffic accident. Pericardial (1.2 mm), left pleural (20 mm) and peritoneal effusion were identified. Inflammatory markers were markedly raised (PCR 271 mg/l and VSG 108 mm/h) whilst full blood count and biochemistry were normal (except Hb 10.4 g/l). Serology (viral and bacterial) and immunology screening remained negative as was genetic study testing for 12 genes for MEFV.

The diagnosis of idiopathic pericarditis was made and treatment with oral corticosteroids (2 mg/kg) for 6 weeks resulted in a good clinical response; however symptoms reoccurred repeatedly when steroids were tapered even though colchicine was added. When immunomodulatory therapy with Il-1R antagonist anakinra (2 mg/kg/day) was initiated, steroids could be withdrawn successfully. Then he suffered benign transitory intracranial hypertension. Treatment with anakinra was reduced after 10 months to 2m/kg alternating days and successfully stopped in February 2011. He remains clinically asymptomatic with normal inflammatory markers.

## Conclusions

1. Idiopathic recurrent pericarditis might be considered an autoinflammatory syndrome.

2. Immunomodulation with Anakinra should be considered in the management of these patients who do not maintain long term response using conventional treatment.

